# Diurnal Changes of Zooplankton Community Reduction Rate at Lake Outlets and Related Environmental Factors

**DOI:** 10.1371/journal.pone.0158837

**Published:** 2016-07-08

**Authors:** Robert Czerniawski, Łukasz Sługocki, Monika Kowalska-Góralska

**Affiliations:** 1 Department of General Zoology, University of Szczecin, Szczecin, Poland; 2 Centre of Molecular Biology and Biotechnology, University of Szczecin, Szczecin, Poland; 3 Department of Hydrobiology and Aquaculture, Wrocław University of Environmental and Life Sciences, Wrocław, Poland; University of Shiga Prefecture, JAPAN

## Abstract

The reduced zooplankton abundance at the outlet sections of lakes depends on the occurrence of preying fry. Therefore, light conditions can play a major role in the drift of zooplankton along river outlets. The aim of this study was to determine the influence of diurnal light conditions on the decline of zooplankton densities at lake outlets. Photosynthetically active radiation (PAR) levels were measured to determine their effect on the rate of reduced zooplankton abundance. Cladocerans and copepods showed a significantly greater reduction in abundance than rotifers and nauplii. A significant positive relationship was observed between the PAR levels and the reduced abundance of *Asplanchna* sp., small cladocerans, large cladocerans and Copepoda at the lake outlets. Among the rotifers, small pelagic rotifers drifted the farthest at all hours of the day. Large crustaceans, especially the large cladocerans and copepodites and adult copepods, had the lowest chance of dispersing over a wide area. Our results indicate that light conditions play an important role in the reduction of zooplankton abundance at lake outlets and have an indirect influence on the downstream food web.

## Introduction

Flow-through lakes and reservoirs provide a rich source of zooplankton for river ecosystems. At lake outlets, zooplankton assemblages reach high densities with high biomasses because the organisms wash out of the upper sections of lakes into the river outlets. Because of this high abundance, high densities of fry that prey on plankters at lake outlets are observed, especially cyprinid fish [1], which make the most significant contribution to the reduction in zooplankton communities [2–6]. The largest plankters, cladocerans, show the greatest reductions. The highest reductions occur in the first 0.2 km downstream section from the lake outlet [1, 7, 8]. In further river sections, the main components of the drifting zooplankton are small rotifers and the nauplii of Cyclopoida.

The high abundance of zooplankton at lake outlets depends on the temporal migration of zooplankton in lakes and thus on diurnal changes in the light conditions. It is generally known that during daytime hours, the zooplankton assemblages migrate to deeper and darker sections of lakes to avoid predators, whereas at night, these assemblages migrate to upper sections to find better food conditions [2, 9, 10], and a similar pattern is observed in deep running waters [11, 12]. Therefore, at lake outlets, much higher abundances of zooplankton are observed at night than during the day. Thus, if the fry that prey on zooplankton find and catch plankters by sight, then light conditions would play a central role in the drift of zooplankton through lake outlets, especially in the first 0.2 km section. However, Czerniawski and Domagała [1] did not observe significant correlations between the decline in zooplankton density at lake outlets and light conditions; however, this study was only performed during the day, and the authors claimed that each value of daylight illuminance was sufficient for the fish to see and catch zooplankton and subsequently cause a reduction in the zooplankton communities at the outlet section. Based on this claim and the lack of data on the relationship between light and zooplankton density at lake outlets, it is important to determine the light conditions that increase the drift of zooplankton from outflow lake areas to downstream reaches and the conditions that cause reduced zooplankton abundance in this section because these conditions likely have important and indirect impact on downstream trophic conditions. These data may be even more important because of the significance of riverine zooplankton in the downstream food web. Moreover, a number of studies have described the pattern of reduced zooplankton abundance in outlet sections [1, 7, 8, 13, 14, 15]; however, none of these studies have shown how light conditions indirectly influence this reduction.

We are aware that other environmental variables, such as discharge, current velocity or suspended solids, can also have an impact on the reduction in zooplankton communities at lake outlets [1, 8, 15, 16, 17]. As the discharge, current velocities or suspended solids in the outlet sections increase, the observed reduction in zooplankton communities decreases. Therefore, these variables can hinder or facilitate plankter predation by fish. Nevertheless, the light conditions can be one important factor affecting the distance zooplankton drift downstream from the outflow.

The aim of this study was to determine the influence of diurnal light conditions on the decline of zooplankton densities at lake outlets.

## Methods

**Fig 1:**
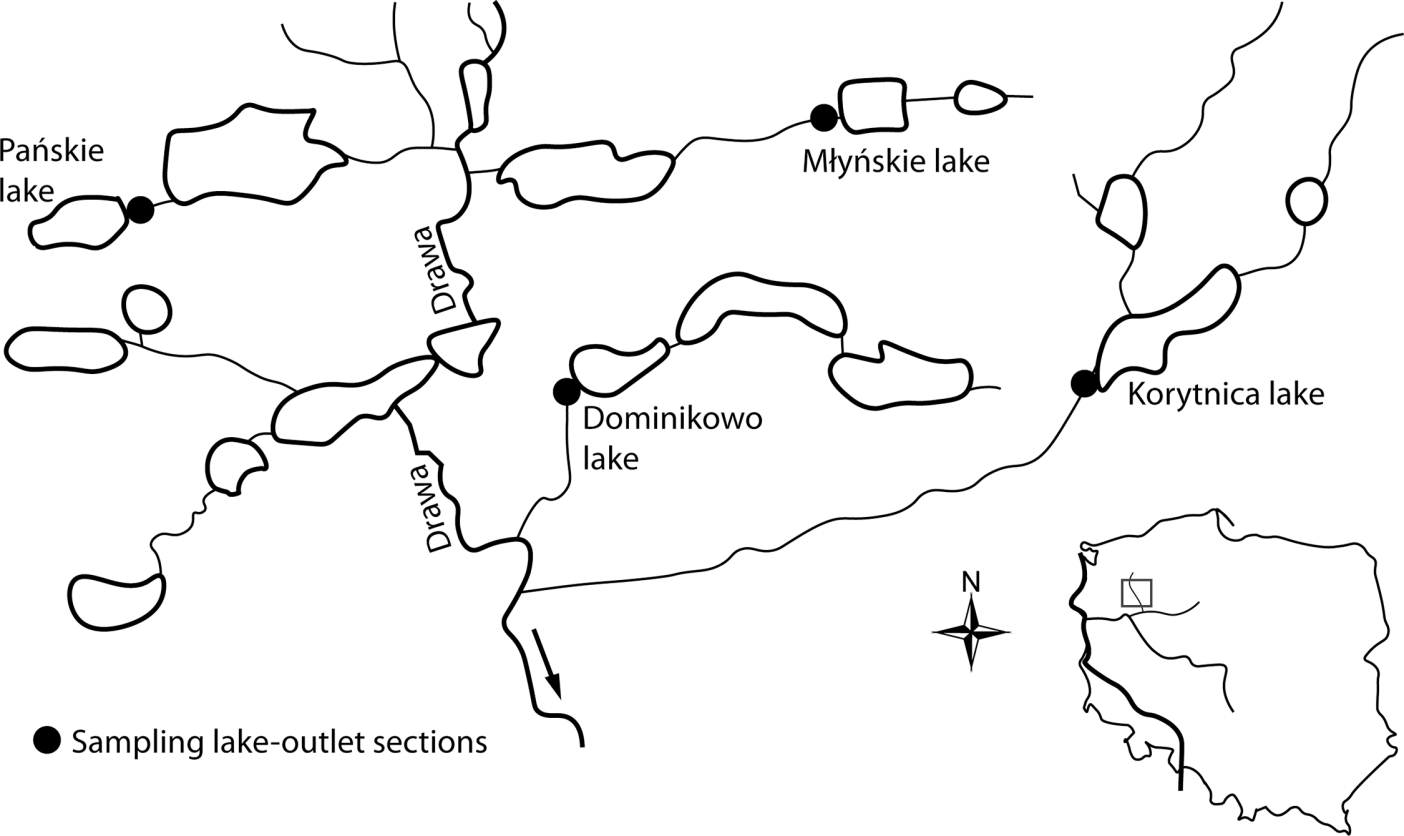
Study area.

The study was performed at the outlet sections of the following four lakes located in the Drawa catchment area (GPS: 53 20 25 N; 15 46 30 E; middle Drawa, drainage for the Oder River, Poland): Lake Pańskie (0.36 m^3^ s^-1^), Lake Młyńskie (0.30 m^3^ s^-1^), Lake Dominikowo (0.27 m^3^ s^-1^), and Lake Korytnica (0.71 m^3^ s^-1^) (Fig 1). At each outlet section, two sampling sites were selected. The first site was directly at the outflow, and the second site was 0.2 km downstream from the outflow. The greatest zooplankton community decline occurred in the 0.2 km long section downstream from the outlet [1, 7, 8]. The zooplankton samples were collected every hour in 27 (lake Dominikowo and Korytnica), 28 (lake Pańskie) and 29 (lake Młyńskie) July 2014. At each site, 50 L of water was collected with a 10 L bucket from the river current, and the samples were then concentrated to 150 mL. The water was filtered through a 10 μm mesh net, and the samples were then fixed in a 4–5% formalin solution. Using the stirred total sample, five sub-samples (2 mL) were pipetted into a glass Sedgewick-Rafter Counting Chamber. The samples were identified using a Nikon Eclipse 50i microscope. The zooplankton was divided into seven groups according to their body size and environmental preferences [18, 19]: i) benthic rotifers (Bdelloidea, Colurellidae, *Mytilina* sp., *Euchlanis* sp., *Lecane* sp., *Cephalodella* sp.), ii) pelagic rotifers (*Pompholyx* sp., Filinidae, Trichocercidae, Brachionidae, *Polyarthra* sp., *Synchaeta* sp.), iii) *Asplanchna* sp., iv) small cladocerans (Bosminidae, Chydoridae), v) large cladocerans (Dapnidae, *Diaphanosoma* sp.), vi) copepod nauplii and vii) copepods (adult copepods and copepodites) were sampled.

At each outlet section, the qualitative composition and biomass of the fish were determined. To ensure that the composition of the fish at the outlet section was the same at night and during the day, the outlet section was separated with two gill nets the day prior to the collection of zooplankton, with one gill net placed at the outflow and the other gill net placed 200 m downstream. The mesh size of the net was 5.0 mm, and the net extended from the bottom of the stream to 1 m above the water surface. A similar procedure was used in another stream experiment [20]. The section between the nets constituted the outlet section. From the stream to the lake outflow section, the zooplankton samples were collected at the outflow 2 m below the net and downstream 2 m before the net. The nets were cleaned sporadically because only slight net clogging with flowing objects or plant parts was observed. The fish were caught along the entire outlet section from the outflow section to the downstream site using electric fish gear with pulsed direct current (Hans Grassl ELT60 II, Germany). Fish samples were caught during the day after zooplankton collection because fish collection on the day of zooplankton collection or beforehand could cause stress to the fish and an improper zooplankton reduction that is not representative of natural conditions. To ensure that the majority of fish were caught, the procedure was performed twice. The fish samples were collected by three people, with two people collecting the fish and the third person walking 20 m behind to ensure that any remaining stunned fish were not carried down the river. Then, the fish were moved to cages, measured, and weighed. The fish species (live fish) were separated into different cages, the total biomass of each species was measured, and the total body length of 100 individuals from each species was measured. If the number of individuals representing a given species was lower than 100, then the body lengths of all individuals were measured. After the measurements, the fish were released back into the stream. The list of fish, their biomasses, and the total body length means are shown in Table 1. No special field sampling permits for sites locations, e.g. for national park or other protected area were necessary. We confirm that the owner of the land (Polish Angling Association) gave permission to conduct the study on examined sites. Approvals for use of electric fish gear were not necessary. According to Polish Law on Inland Fisheries the electrofishing with pulsed direct current is permitted by owner of farm fishing lake. All fish catches were conducted with the farm fishing lake owner. Therefore no specific permissions were required for these locations and activities, and therefore no approval of Institutional Animal Care and Use Committee (IACUC) or equivalent animal ethics committee provide details on why this is the case was not required. The field studies did not involve endangered or protected species. All sampling procedures and experimental manipulations required for our study were reviewed or specifically approved as part of obtaining the field permit. All fish survived the manipulation.

**Table 1:** Total wet biomass (g) and mean total body length (cm) of fish species caught in the outlet sections. The body length values are shown in parentheses.

Fish species	Pańskie	Młyńskie	Dominikowo	Korytnica
**Roach *(Rutilus rutilus)***	3696 (11.5)	2863 (12.3)	2295 (11.8)	1042 (11.5)
**Bleak *(Alburnus alburnus)***	56 (7.5)	623 (8.2)		9753 (9.3)
**Silver bream *(Abramis bjoerkna)***	1423 (12.6)	871 (12.4)	622 (13.5)	486 (12.8)
**Chub *(Gobio gobio)***	236 (7.7)	748 (7.2)	28 (9.3)	17 (5.8)
**Rudd *(Scardinius erythroptalmus)***	1071 (13.8)		106 (17.5)	67 (12.5)
**Bream *(Abramis brama)***	24 (10.4)	58 (12.6)	15 (11.6)	105 (10.8)
**Bitterling *(Rhodeus sericeus)***	34 (4.5)	12 (4.4)		52 (5.3)
**Perch *(Perca fluviatilis)***	886 (10.5)	563 (8.6)	326 (10.9)	123 (11.4)
**Ruffe *(Gymnocephalus cernua)***		25 (12.8)		
**Pike *(Esox lucius)***	68 (13.9)	163 (17.5)	311 (28.5)	115 (24.5)
**Burbot *(Lota lota)***		55 (12.1)	15 (11.0)	

The water velocity, width and depth were measured at 20 m intervals from the outflow site to the downstream site by an electromagnetic water flow sensor OTT (Germany) to determine the water discharge. The average velocity was calculated from the velocities measured in three sections across the stream at depths of 20% and 80%. The content of suspended solids was measured at day in the outflow by a photometer DR-850 (Hach Lange, USA). We visually estimated the total percentage of vegetation cover along the entire outlet section. The results of the above variables in each outlet section are shown in Table 2.

**Table 2:** Values of the basic characteristics of the examined outlet sections.

Site	Vegetation cover (%)	Suspended solids (mg l^-1^)	Velocity (m s^-1^)	Width (m)	Depth (m)	Discharge (m^3^ s^-1^)
**Pańskie**	2	21	0.30	6.00	0.20	0.36
**Młyńskie**	12	10	0.29	4.10	0.25	0.30
**Dominikowo**	25	3	0.22	4.25	0.29	0.27
**Korytnica**	30	18	0.29	6.10	0.40	0.71

To determine the influence of the volume of light on the abundance of zooplankton in the outflow, the vertical migration of zooplankton and their washout into the outflow, the illuminance (lux) was measured before the outflow above the surface of the lakes to demonstrate that zooplankton from the upper strata of the lake reach the outflow because of diurnal migrations that depend on the volume of illuminance. An analysis of the illuminance results and zooplankton abundance was performed to determine the optimal time of day and amount of illuminance for the collection of zooplankton at lake outflows. Photosynthetically active radiation (PAR) was measured at 20 m intervals from the outflow site to the downstream site to determine the effect of PAR on the reduction in zooplankton abundance. Illuminance was measured by a lux metre (Sonel LXP-1), whereas the PAR was measured by a multiparameter sonde (Hydrolab DS5x). The values of the PAR and illuminance (lux) are shown in Table 3, and these two parameters were significantly correlated in each outlet section (Spearman test, *r* = 0.90, P < 0.05).

**Table 3:** Values of illuminance (lux) and PAR (μmol photons m^−2^ s^−1^) of study outlet sections.

	Pańskie		Młyńskie		Dominikowo		Korytnica	
Hour	Illuminance	PAR	Illuminance	PAR	Illuminance	PAR	Illuminance	PAR
**12.00**	6557	155	8542	319	8870	407	7863	197
**13.00**	6522	138	8657	343	5310	275	7638	171
**14.00**	6343	122	7246	292	4370	151	6425	163
**15.00**	5473	127	7054	235	4010	140	6213	161
**16.00**	4228	88	5575	212	3260	115	5237	124
**17.00**	2751	64	3765	145	2590	105	3478	84
**18.00**	2004	48	3421	128	2090	96	3041	71
**19.00**	955	23	1534	116	940	56	1423	54
**20.00**	118	17	467	23	390	17	550	21
**21.00**	22	2	43	2	0.1	0	58	3
**22.00**	0	0	0.1	0	0	0	0.1	0
**23.00**	0	0	0	0	0	0	0	0
**0.00**	0	0	0	0	0	0	0	0
**1.00**	0	0	0	0	0	0	0	0
**2.00**	0	0	0	0	0	0	0	0
**3.00**	0	0	0	0	0	0	0	0
**4.00**	0.05	0	0.2	0	0.01	0	0.4	0
**5.00**	26	1	24	1,12	0,08	0	31	1
**6.00**	305	15	2091	88,2	850	44	1476	58
**7.00**	2843	54	4325	99,4	3890	137	4581	79
**8.00**	3875	68	5567	187,6	5200	211	5886	152
**9.00**	4467	73	6230	203	5800	262	6458	165
**10.00**	5881	127	8232	264,6	7580	378	7743	186
**11.00**	6754	175	8458	312,2	9670	634	8883	248

Each of the four outlet sections was treated as a separate repetition. All of the mean values of diurnal zooplankton abundance and diurnal reduction of zooplankton abundance shown on the figures are the sum of the sampled values divided by the number of items of all four outlet sections combined. Moreover mean abundance and mean reduction rates of abundance between each outlet section were compared. For each outlet section a correlation between illuminance values and the abundance of zooplankton in outflow and also between PAR and the abundance reduction rates of zooplankton were checked. Percentage reduction of the zooplankton abundance was estimated as the difference between the values of abundance in outflow and downstream. The difference result was a percentage abundance value in the outflow.

For checking the significant difference of abundance and values of abundance reduction in each zooplankton group among the sites the Kruskal-Wallis test was used (P < 0.05). For determining the significant differences between sites and lake outlet sections in abundances and values of abundance reduction the post-hoc multiple comparisons of mean ranks for all groups was done (P < 0.05). The correlation between illuminance values and the abundance of zooplankton in outflow and the relationships between PAR and the abundance reduction rates of zooplankton were checked using Spearman’s rank correlation.

## Results

### Diurnal abundance of the zooplankton in the outflow

**Fig 2:**
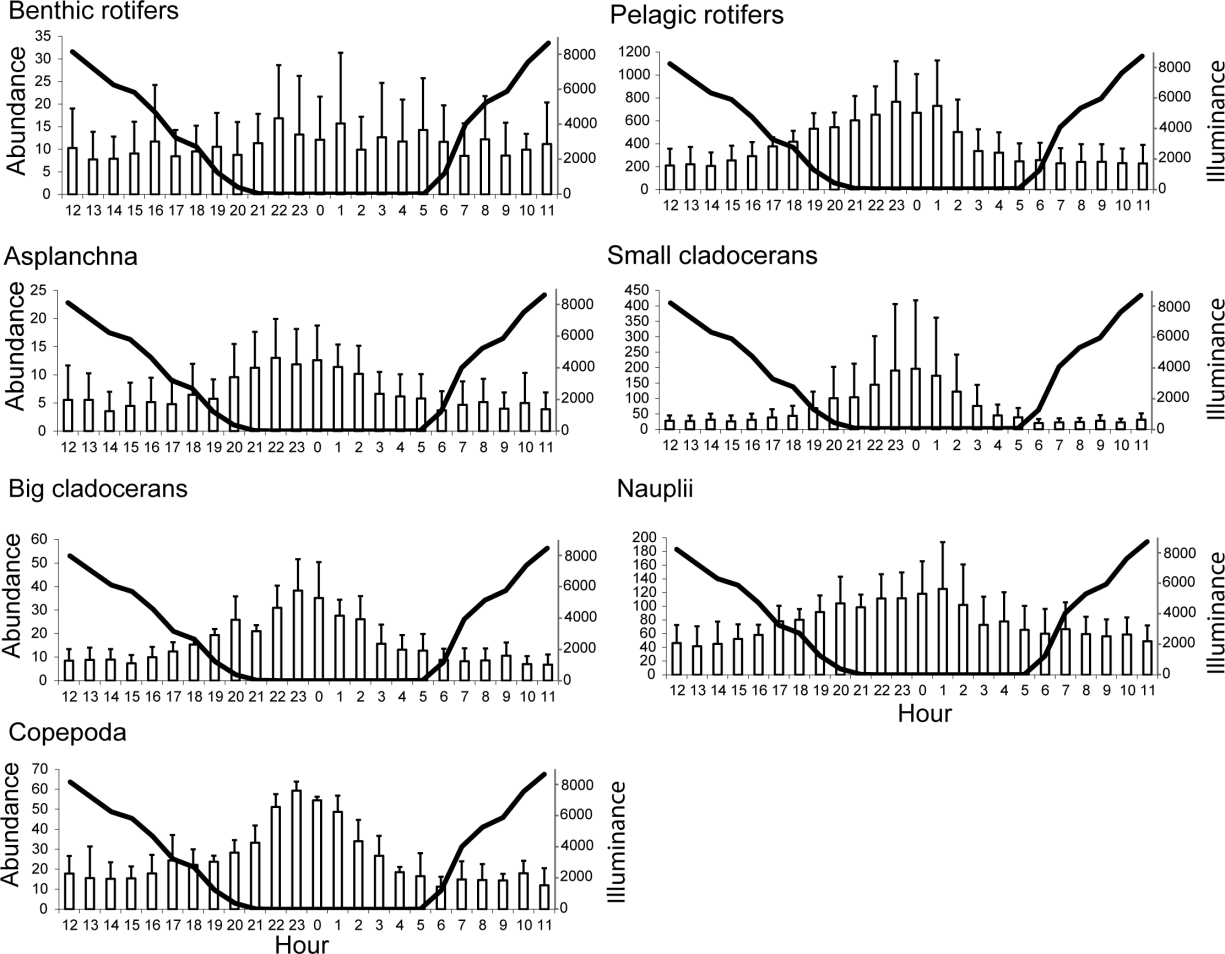
Mean + SD of the zooplankton abundance (ind. l^-1^) in the outflows of the examined lakes in relation to the mean illuminance values (lux) (solid line).

The range of mean abundance for the zooplankton in the outflow was as follows: benthic rotifers, 8 to 11 ind. L^-1^; pelagic rotifers, 207 to 766 ind. L^-1^; *Asplanchna* sp., 4 to 13 ind. L^-1^; small cladocerans, 20 to 196 ind. L^-1^; large cladocerans, 7 to 38 ind. L^-1^; nauplii, 42 to 125 ind. L^-1^; and copepods, 11 to 59 ind. L^-1^ (Fig 2). In the case of benthic rotifers, the abundance was relatively constant at all hours, whereas all of the other groups presented relatively constant abundances from 06:00 to 16:00 during the day at mean illuminance values of 1180 to 4575 lux.

Subsequently, none of the zooplankton groups presented significant differences in their mean outflow abundance (P > 0.05), which indicates that differences in the increasing and declining abundance trends in the outflow for this time period proceeded rather smoothlyoccurred gradually.

### Mean abundance of the zooplankton

In the outflow and downstream sections of each lake outlet section, pelagic rotifers showed a the highest mean abundance (Table 4). In each outlet section with the exception of Pańskie lake the benthic rotifers presented a higher abundance downstream than in the outflow (S1–S4 Tables), however the differences (reduction rates) were small. The abundance of other groups was significantly higher outflow than in the downstream. The outlet section of Dominikowo lake characterized by lowest values of the zooplankton abundance. The abundance of each group of zooplankton in the outflows showed higher values with the decline in the illuminance values. Significant negative relationships between illuminance values (lux) and the abundance of all groups of zooplankton, except benthic rotifers, were observed in the outflow of each lake (P < 0.05).

**Table 4:** Mean ± SD of the abundance (ind. l^-1^) in the studied sites of outlet section lake. Different letters in the same row indicate significant differences among zooplankton groups between lake outflows (P < 0.05).

Zooplankton group	Pańskie	Młyńskie	Dominikowo	Korytnica
**Outflow**				
**Benthic rotifers**	24.0±7.3 a	10.7±5.4 a	2.1±1.4 b	7.1±2.6 ab
**Pelagic rotifers**	480.7± 8.2 a	397.9±295.9 a	147.5±125.3 b	528.6±325.7 a
**Asplanchna**	11.9±2.4 a	8.3±4.8 a	0.6±1.2 b	6.9±6.0 a
**Small cladocerans**	49.2±15.5 a	169.1±177.3 b	2.2±1.7 c	51.9±40.2 a
**Large cladocerans**	17.9±6.4 a	16.9±14.5 a	9.2±10.5 b	20.5±11.8 a
**Nauplii**	57.0±9.8 a	85.9±39.5 ab	47.5±38.3 a	114.7±41.9 b
**Copepoda**	19.6±8.4 a	17.3±9.5 a	7.7±8.2 b	56.7±33.3 c
**Downstream**				
**Benthic rotifers**	22.7±7.8 a	11.9±4.5 a	3.7±1.7 b	17.0±6.2 a
**Pelagic rotifers**	434.0±56.8 a	351.2±265.4 a	129.1±110.0 b	483.0±311.3 a
**Asplanchna**	8.2±2.1 a	5.7±4.2 a	0.4±0.9 b	5.7±5.4 a
**Small cladocerans**	20.9±16.4 ab	11.1±150.1 b	0.8±1.0 c	33.4±5.9 a
**Large cladocerans**	6.9±6.8 a	8.3±12.1 a	2.9±4.3 b	9.9±9.9 a
**Nauplii**	48.8±9.7 a	70.9±32.6 ab	40.1±33.1 a	98.6±34.8 b
**Copepoda**	7.9±8.6 a	7.9±8.9 a	3.6±5.3 b	26.5±28.1 c

### Mean reduction of the zooplankton abundance

The highest reduction in abundance was observed for the large cladocerans and copepods and slightly lower rates were observed for the small cladocerans (Table 5). Generally, the outlet of Lake Korytnica was characterized by the lowest mean reduction in zooplankton abundance. The rates of reduction of benthic rotifers in the outlet sections of Lake Pańskie and Lake Młyńskie were significantly higher than that of the outlet sections of Lake Dominikowo and Lake Korytnica (Table 5). The reduction in the mean abundance of pelagic rotifers was similar at each outlet, although the lowest rate was observed at the Korytnica outlet. The mean abundance of *Asplanchna* sp. reached a significantly lower value at the Korytnica outlet relative to the other lake outlets. Cladocerans showed the largest decline at the Dominikowo outlet and the lowest decline at the Korytnica outlet. The mean abundance of nauplii and copepods was similarly reduced at each outlet.

**Table 5:** Mean ± SD of the zooplankton abundance reduction (%) in outlet section (200 m) of study lakes. Different letters in rows indicate significant differences between lake outlets (P < 0.05).

Zooplankton group	Pańskie	Młyńskie	Dominikowo	Korytnica
**Benthic rotifers**	3.8±21.2 b	-26.9±63.4 b	-126.2±108.4 a	-148.7±75.0 a
**Pelagic rotifers**	9.8±3.1 ab	12.4±3.1 a	13.0±2.9 a	9.6±3.5 b
**Asplanchna**	31.9±8.9 b	36.3±17.1 ab	65.0±34.6 a	21.5±11.3 c
**Small cladocerans**	60.8±22.1 a	51.8±22.1 b	69.0±30.5 a	46.8±17.5 b
**Large cladocerans**	68.0±24.8 ab	70.3±29.6 ab	74.7±28.4 a	60.6±22.6 b
**Nauplii**	14.7±3.4 a	17.5±4.4 b	15.6±7.7 a	13.7±4.6 a
**Copepoda**	66.6±23.3 a	65.5±25.2 a	68.2±24.8 a	62.2±21.2 a

### Diurnal reduction of the zooplankton abundance

**Fig 3:**
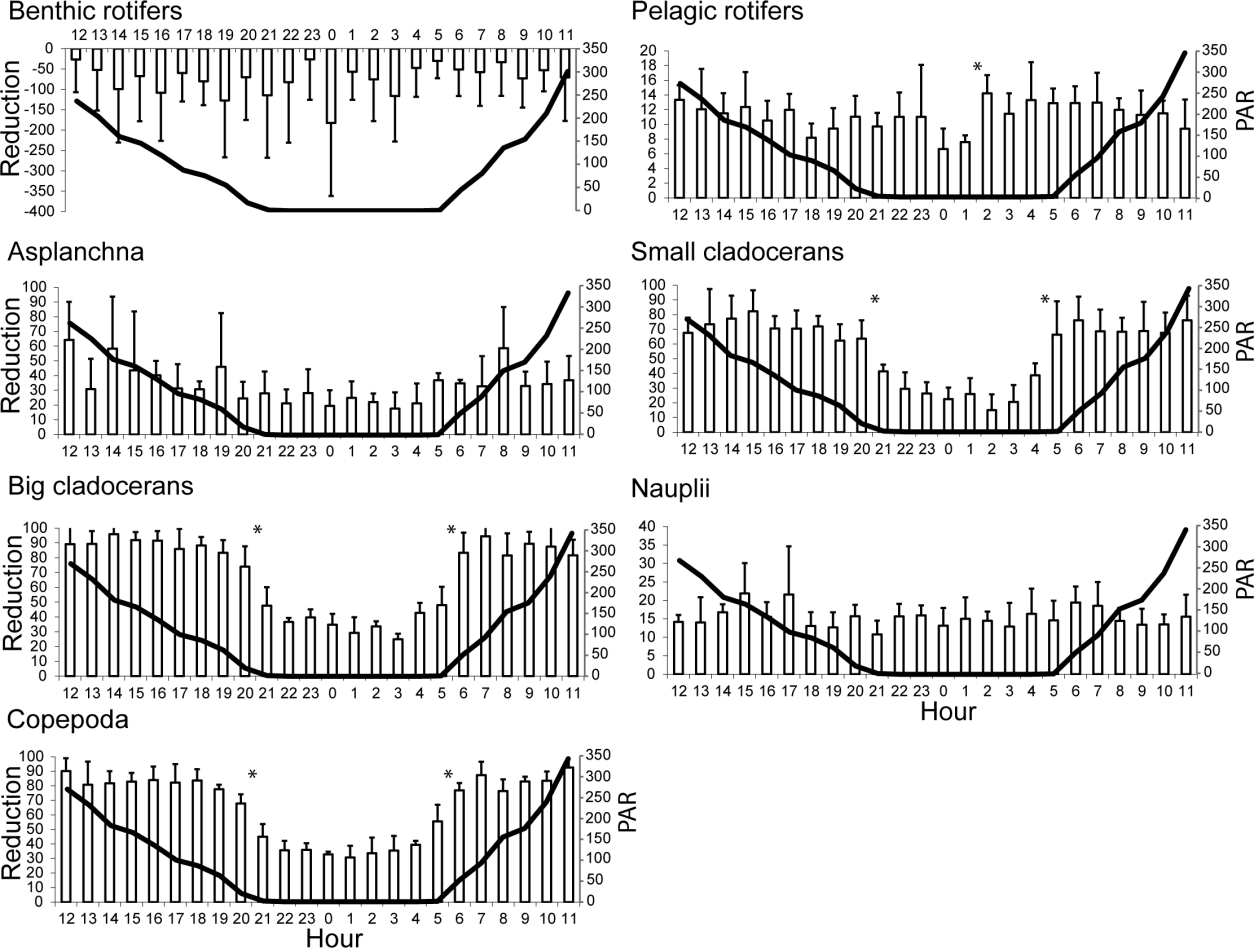
Mean + SD of the diurnal reduction in zooplankton abundance (%) in the outlet sections of the examined lakes in relation to the mean PAR values (μmol photons m^−2^s^−1^) (solid line). * P < 0.05, significant differences in the reduction rates for the subsequent hours.

The mean reduction ranges of zooplankton were as follows: benthic rotifers, -182 to -126% (P > 0.05); pelagic rotifers, 7 to 14% (P > 0.05); *Asplanchna* sp., 18 to 64% (P > 0.05); small cladocerans, 15 to 82% (P < 0.05); large cladocerans, 25 to 96% (P < 0.05); nauplii, 11 to 22% (P > 0.05); and copepods, 31 to 93% (P < 0.05) (Fig 3). For rotifers and nauplii, the reduction in abundance was relatively constant at all hours, whereas for adult crustaceans, the reduction was relatively constant from 06:00 to 20:00 at mean PAR values of 51–20 μmol photons m^−2^s^−1^.

Subsequently, benthic rotifers, pelagic rotifers, *Asplanchna* sp. and nauplii did not show significant differences in the rate of reduced mean abundance (P > 0.05) except for an increase in the reduction of pelagic rotifers (50%) between 01:00 and 2:00 (P < 0.05). Small cladocerans showed a significant decline in abundance (35%) between 20:00 and 21:00 (P < 0.05) and a significant increase (41%) between 4:00 and 5:00 (P < 0.05). Large cladocerans and copepods showed significantly declined and significantly increased reductions in abundance between 20:00 and 21:00 (36% and 34%, respectively) and 05:00 to 06:00 (42% and 29%, respectively) (P < 0.05).

**Fig 4:**
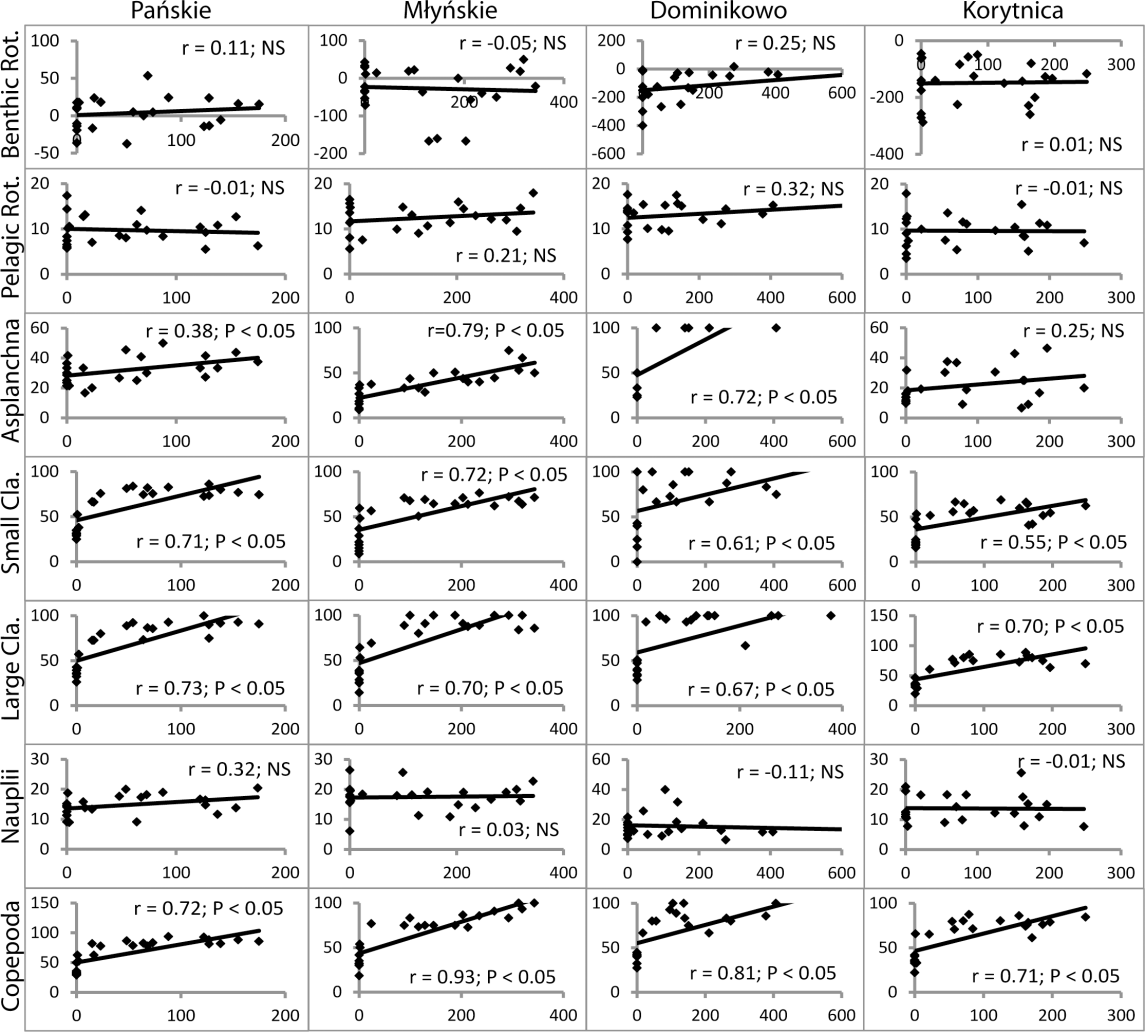
Relationship between PAR (μmol photons m^−2^s^−1^) and abundance reduction (%) of the zooplankton groups (ind. l^-1^) in examined lake outlets. r–index of Spearman’s rank correlation, NS–not significant, Benthic Rot.–benthic rotifers, Pelagic Rot.–pelagic rotifers, Small Cla.—small cladocerans, Large Cla.–large cladocerans.

Reduction rates in the abundance of all of the zooplankton groups corresponded to values of PAR. A significant positive relationship was observed between PAR and the reduced abundance of *Asplanchna* sp. (with the exception of Korytnica lake P > 0.05), small cladocerans, large cladocerans and copepods at the outlets (P < 0.05) (Fig 4).

## Discussion

The zooplankton assemblage in lakes presents a vertical distribution and diel vertical migration in reaction to light conditions, predator occurrence or temperature and oxygen conditions [2, 9, 10, 21]. With regard to these migrations, a higher abundance of zooplankton is observed in the upper sections of lakes at night than during the day. It is also generally known that zooplankton is washed out of lakes by stream or river outflows. One hypothesis is that in stagnant basins, the outflow pattern corresponds to the vertical migration of zooplankton in lakes [15]. Therefore, as the abundance of zooplankton in the upper zones of lakes increases, the density of zooplankton in the outflow increases. In the examined outflows, a typical diurnal abundance of zooplankton in the upper zones of the lakes was observed. As expected, the abundance of zooplankton in the outflows was strongly negatively correlated with the volume of illuminance over the water surface. Hence, it is not surprising that the abundance of zooplankton in the outflow at night was much higher than that during the day.

The reduced zooplankton abundance at the outlet sections of lakes may be caused directly by preying fish, preying macro-invertebrates, mechanical damage or sedimentation [7, 15, 17, 21], and declining zooplankton communities at the outlet sections of lakes or reservoirs are mostly related to the presence of fry in these areas [7, 8, 22]. The cited authors reported that the largest plankters, daphnids, and adult Copepoda are the first to undergo reductions, and then the smaller Cladocera experience reductions, with rotifers and nauplii the last to be reduced. The cause of this pattern is the size selection of zooplankton by fish, and it is cited as one of the main factors that determines the structure of crustaceans, particularly daphnids and adult copepods, in flowing water [15, 22, 23]. However, fish do not have a negative effect on the composition of rotifers [7, 14, 24]. Similar patterns were observed during the day in the outlet sections of the present study. The effectiveness of fish foraging on zooplankton depends on many environmental factors. Wissel *et al*., [25] claimed that a smaller abundance of zooplankton may be caused by increased water transparency, which facilitates predation by fish. The results of the present study on the effectiveness of zooplankton foraging by fish in the outlet section were also indirectly dependent on the light conditions. The effect of the positive influence of the light volume on fish foraging skills has been described in several papers [2, 26].

The significant reduction in the communities of small cladocerans, such as bosminids and chydorids, by fish predation is not consistent with the results of several experimental studies in which the presence of fish was not found to have a negative effect on the density of *Bosmina* sp. or *Diaphanosoma* sp. [27, 28]. However, Jack and Thorp [6] claimed that a low encounter rate between predators and bosminids or certain daphnids and the relatively high densities of copepods may have resulted in the fish focusing their foraging efforts on the more abundant copepod prey. Hence, it should be assumed that the influence of fry on the communities of small cladocerans is also significant, especially when the latter occur in high densities, which has been confirmed by other authors.

Because of the shallow depth of the examined streams, the increased contact with stream sediments in the shallow sections, which present higher flow velocities, could also cause the decreased zooplankton abundance downstream of the lakes, and a similar supposition has been noted [29] for the reduced zooplankton density downstream of small dams. Chang *et al*., [7] also claimed that the sedimentation of zooplankters causes a reduction in their abundance at outlet sections, although predation by juvenile fish is a more significant cause of this decline. Grazing by suspension-feeding, filter-feeding or net-spinning macrozoobenthic organisms could also explain the reduction in zooplankton abundance at outlet sections. However, the impact of macrozoobenthos on zooplankton communities is rather small [7, 30, 31, 32]. Thorp and Casper [28] showed that invasive zebra mussels can significantly reduce the abundance of zooplankton. However, in the examined outlet sections, these bivalves were absent or only present in low densities. Thus, the main reason for the reduced zooplankton density downstream of the lakes appears to be fish predation [1, 22, 28].

Compared with the pelagic rotifers or other zooplankton groups, the benthic rotifers showed higher mean abundances in the downstream sections than in the outflow sections. Therefore, their density was not reduced but increased at the outlet sections, and this pattern was frequently observed. Benthic rotifers and bdelloids (70% of the abundance of the observed benthic rotifers) are associated with benthic or littoral zones covered by macrophytes [32–34]. Therefore, the high abundance of benthic rotifers in the outlet sections may have been related to the strength of the water current because greater amounts of benthic rotifers, especially bdelloids, are observed in streams with water currents strong enough to transport these organisms away from the bottom or the macrophytes [34, 35]. The examined outlet sections of lakes Dominikowo and Korytnica had the greatest coverage of macrophytes, which could be a source of benthic rotifers in the water. The reduction of other zooplankton groups differed depending on the environmental parameters of the outflow. Authors have indicated that high discharge, current velocity or depth values and transparency values promote the wide-spread dispersal of all plankters [1, 15, 16, 35, 36]. As the discharge, depth and current velocities increase, the dispersal distance of zooplankton also increases. The present study indicates that the lowest reductions occurred in the widest and deepest sections, and lower reductions were also observed in areas with the highest concentration of suspended solids. In addition, the results indicate that the influence of fish biomass on the reduction in zooplankton abundance is depended on the environmental factors of outlets. For example, in the outlet section of Korytnica, the fish biomass, especially that of cyprinids, was highest and the reduced abundance was lowest, whereas in the outlet section of Dominikowo, the fish biomass was the lowest and the reduced zooplankton abundance was highest. Therefore, these results indicate that fish predation causes the greatest reduction in zooplankton abundance and the morphometric conditions of the streambed as well as the transparency and illuminance conditions have also important effects on the feeding efficiency of fish.

Allan and Castillo [37] and Hieber *et al*., [38] found that a significantly higher number of macroinvertebrates occur in the drift of streams at night than during the day. A similar pattern is observed for zooplankton communities in the stream drift. At night, the abundance of cladocerans, copepods and rotifers is much higher than during the day, which suggests a diel vertical migration [11, 12]. Hence, we can conclude that in the outlet sections of lakes, the abundance of zooplankton drifting into the stream from the benthic zone (or the macrophytes area) or slack water areas is increased at night [11, 12, 36, 39, 40]. Additionally, except for the benthic rotifers, the same species were observed in the downstream and outflow sections.

In the present study, the diurnal reduction of particular groups of zooplankton varied. The abundance of the smallest pelagic rotifers and nauplii was reduced by 7–22% each hour, and the rates were independent of the PAR values. This result indicates that one variable can may primarily affect the reduced abundance. For example, if the fish ignored or did not see the small drifting plankters, then the main reason for the zooplankton reduction was most likely sedimentation or macro-invertebrate feeding. Nevertheless, the reduced abundance of small plankters was insignificant, whereas the reduction of small and large cladocerans and copepodites associated with adult copepods was significant during the day. The lowest significant rate of reduction during the day was for small cladocerans at 82%, whereas the highest was for large cladocerans at 96%. Similar percentage reductions were recorded by Czerniawski and Domagała [1, 8]. for the same groups. During evening, night and early morning hours when the PAR ranged from 20 to 1 μmol photons m^−2^ s^−1^, the lowest reduced abundance of cladocerans and copepods ranged from 15 to 31%. Therefore, during the day, the reduced abundance of crustaceans was primarily caused by predation by juvenile or adult fish [17], whereas at night, the reduced abundance of crustaceans was caused by sedimentation, macroinvertebrate predation, or physical damage [7]. Therefore, the diurnal reduction of small pelagic plankters and nauplii was small, similar over each hour, and primarily caused by sedimentation, physical damage or macroinvertebrates predation during the day and at night, whereas the reduction in copepodites and adult crustaceans varied according to the light conditions. During the day, the reduction in copepodites and adult crustaceans is primarily caused by fish predation and to a lesser extent by sedimentation, physical damage or macro-invertebrates predation, whereas at night, this reduction is most likely only caused by sedimentation, physical damage or macroinvertebrate predation. Based on our results, we can conclude that the reduction in the abundance of all zooplankton groups was similar at night, whereas during the day, the reduction of *Asplanchna* sp., copepodites and adult microcrustaceans was much higher than that of pelagic rotifers and nauplii. However we feel that the densities of *Asplanchna* sp., in both outflow and downstream are too low to show clearly the reduction.

Czerniawski and Domagała [1] did not observe significant correlations between the reduced biomass of zooplankton and the volume of illuminance during the day at lake outlets, and they concluded that the daily volume of illuminance independent of the volume of cloud cover or shading is sufficient for fish to feed on zooplankton. These authors also found a strong positive correlation between the reduced biomass of adult crustacean and the density of cyprinids. Based on their results, the relationship between the zooplankton abundance at the outflows and the reduced abundance at the outlet sections were noted in the present study. The increased mean abundance of zooplankton in the outflows started at 17:00; however, significant reductions in the abundance of crustaceans ended at 20:00. This result indicates that with the decreased illuminance after 17:00, the pelagic plankters and crustaceans migrated to the upper sections of the examined lakes and were washed to the outflows. However, the volume of illuminance or the PAR from 17:00 to 20:00 was sufficient for fish to observe the plankters, especially the adult crustaceans. Similar patterns have been described by Gliwicz [2] in the African lake Cohora Bassa during evening twilight hours before moonrise when the food migration of plankters to the upper sections is most intense and an intense increase of preying planktivorous sardines is observed. Sardines feed on zooplankton most efficiently at night when a full or nearly full moon rises after sunset because zooplankton approach the surface during darkness and become suddenly vulnerable in the first light of the rising moon. In the present study, however, similar patterns were not observed at dawn. The declining abundance of zooplankton, adult crustaceans especially in the outflows started at 06:00, which may indicate that the fry had eaten in the evening of the previous day and had hidden themselves from other fish predators at twilight. As is generally known, fish feeding is most intense in the evening [2].

The results of the present study also show how dispersal depends on the size of the different groups of plankters. Among the rotifers, all of the small pelagic rotifers can drift farthest during both the day and night. Large crustaceans have the lowest chance of wide-spread dispersal, especially the large size cladocerans and copepodites and adult copepods. However, a widespread dispersal of copepods may be possible because of the small size of nauplii, which showed insignificant reductions in the outlet section throughout the entire the day. Hence, nauplii can drift downstream and find good conditions for development. Among the crustaceans, copepods are most often found downstream in lakes or reservoirs [41]. This result may be caused by the dispersal of adult individuals, which is doubtful, or the dispersal of nauplii that subsequently develop in the suitable mesohabitats downstream. Although this assumption is speculative, it should be verified in future studies.

Our results also show that in the analysed lake outflows, the abundance of zooplankton is relatively constant from 06:00 to 16:00 in temperate climates in the summer or under illuminance volumes from approximately 1200 to 5400 lux, and the optimal abundance is sampled between those hours. At conditions that support the mean of these values, the optimal zooplankton samples can be collected.

We are aware that the diurnal reduction in zooplankton at outlets sections can vary in seasons other than the summer. In autumn and winter, the density of fry or preying fish in outlet sections is much lower compared with that in summer, and all of the plankters could disperse farther downstream. A similar pattern to that observed in our study could also be created in spring when fish hatching occurs. Studies have shown that the reduction in zooplankton communities in winter is lower than that in summer [8, 42]. Moreover, the pattern of reduction in zooplankton communities could be different in the outflows of larger streams or large rivers, and the time of day, the season or the morphological conditions of the outlets sections may have different effects on the trophic downstream conditions caused by the dispersion of the zooplankters from associated lakes or reservoirs. As shown in the present study, the light conditions in each season and at each outlet section play an important role in the reduced abundance of zooplankton. However, this effect is indirect.

## Supporting Information

S1 Table Values of light conditions and zooplankton abundance in Pańskie lake outlet. All data used for analysis.
(DOCX)

S2 Table Values of light conditions and zooplankton abundance in Młyńskie lake outlet. All data used for analysis.
(DOCX)

S3 Table Values of light conditions and zooplankton abundance in Dominikowo lake outlet. All data used for analysis.
(DOCX)

S4 Table Values of light conditions and zooplankton abundance in Korytnica lake outlet. All data used for analysis.
(DOCX)
