# Supplementary material for: Diurnal Changes of Zooplankton Community Reduction Rate at Lake Outlets and Related Environmental Factors
Source: PLoS One. 2016 Jul 8;11(7):e0158837. doi: 10.1371/journal.pone.0158837 (PMC4938256; doi:10.1371/journal.pone.0158837)
Supplement: S1 Table — All data used for analysis. (DOCX) [file pone.0158837.s001.docx]

**S1 Table. Values of light conditions and zooplankton abundance in Pańskie lake outlet.** All data used for analysis.

| Hour | Site | Lake outlet | Benthic rotifers  (ind l^-1^) | Pelagic rotifers  (ind l^-1^) | Asplanchna  (ind l^-1^) | Small cladocerans  (ind l^-1^) | Large cladocerans (ind l^-1^) | Nauplii  (ind l^-1^) | Copepoda  (ind l^-1^) | Illuminance  (lux) | PAR  (µmol photons m^−2^ s^−1^) |
| --- | --- | --- | --- | --- | --- | --- | --- | --- | --- | --- | --- |
| 12 | outflow | Pańskie | 25 | 441 | 16 | 39 | 14 | 58 | 17 | 6557 |  |
| 13 | outflow | Pańskie | 18 | 462 | 12 | 45 | 12 | 43 | 22 | 6522 |  |
| 14 | outflow | Pańskie | 14 | 384 | 9 | 40 | 13 | 48 | 14 | 6343 |  |
| 15 | outflow | Pańskie | 21 | 452 | 11 | 36 | 10 | 55 | 17 | 5473 |  |
| 16 | outflow | Pańskie | 33 | 489 | 12 | 52 | 14 | 58 | 16 | 4228 |  |
| 17 | outflow | Pańskie | 18 | 411 | 12 | 47 | 15 | 66 | 23 | 2751 |  |
| 18 | outflow | Pańskie | 16 | 422 | 15 | 43 | 18 | 68 | 15 | 2004 |  |
| 19 | outflow | Pańskie | 22 | 511 | 10 | 62 | 20 | 60 | 18 | 955 |  |
| 20 | outflow | Pańskie | 21 | 555 | 12 | 68 | 22 | 58 | 16 | 118 |  |
| 21 | outflow | Pańskie | 17 | 578 | 14 | 58 | 21 | 67 | 21 | 22 |  |
| 22 | outflow | Pańskie | 29 | 554 | 17 | 78 | 25 | 71 | 37 | 0 |  |
| 23 | outflow | Pańskie | 34 | 581 | 12 | 83 | 28 | 66 | 41 | 0 |  |
| 0 | outflow | Pańskie | 26 | 554 | 14 | 75 | 28 | 77 | 35 | 0 |  |
| 1 | outflow | Pańskie | 42 | 563 | 12 | 66 | 34 | 64 | 31 | 0 |  |
| 2 | outflow | Pańskie | 19 | 484 | 14 | 48 | 25 | 62 | 24 | 0 |  |
| 3 | outflow | Pańskie | 33 | 493 | 10 | 38 | 19 | 53 | 18 | 0 |  |
| 4 | outflow | Pańskie | 27 | 462 | 11 | 41 | 17 | 56 | 14 | 0,05 |  |
| 5 | outflow | Pańskie | 34 | 464 | 12 | 42 | 19 | 48 | 16 | 26 |  |
| 6 | outflow | Pańskie | 18 | 455 | 9 | 21 | 11 | 38 | 11 | 305 |  |
| 7 | outflow | Pańskie | 20 | 397 | 11 | 37 | 13 | 45 | 14 | 2843 |  |
| 8 | outflow | Pańskie | 28 | 468 | 11,5 | 39 | 15 | 52 | 9 | 3875 |  |
| 9 | outflow | Pańskie | 21 | 483 | 8 | 45 | 14 | 44 | 12 | 4467 |  |
| 10 | outflow | Pańskie | 15 | 411 | 14 | 34 | 12 | 61 | 16 | 5881 |  |
| 11 | outflow | Pańskie | 26 | 462 | 8 | 43 | 11 | 49 | 14 | 6754 |  |
| 12 | downstream | Pańskie | 21 | 385 | 9 | 9 | 1 | 50 | 2 |  | 155 |
| 13 | downstream | Pańskie | 19 | 412 | 8 | 9 | 1 | 38 | 4 |  | 138 |
| 14 | downstream | Pańskie | 16 | 344 | 6 | 11 | 0 | 40 | 1 |  | 122 |
| 15 | downstream | Pańskie | 16 | 427 | 8 | 5 | 1 | 46 | 2 |  | 127 |
| 16 | downstream | Pańskie | 25 | 448 | 6 | 9 | 1 | 47 | 1 |  | 88 |
| 17 | downstream | Pańskie | 18 | 366 | 9 | 12 | 4 | 60 | 4 |  | 64 |
| 18 | downstream | Pańskie | 22 | 386 | 11 | 8 | 2 | 56 | 2 |  | 48 |
| 19 | downstream | Pańskie | 18 | 475 | 8 | 15 | 4 | 52 | 4 |  | 23 |
| 20 | downstream | Pańskie | 16 | 482 | 10 | 23 | 6 | 50 | 6 |  | 17 |
| 21 | downstream | Pańskie | 14 | 517 | 11 | 36 | 9 | 61 | 10 |  | 2 |
| 22 | downstream | Pańskie | 33 | 513 | 13 | 51 | 16 | 63 | 26 |  | 0 |
| 23 | downstream | Pańskie | 28 | 543 | 9 | 54 | 17 | 56 | 28 |  | 0 |
| 0 | downstream | Pańskie | 31 | 522 | 11 | 52 | 16 | 70 | 24 |  | 0 |
| 1 | downstream | Pańskie | 38 | 516 | 8 | 47 | 23 | 55 | 22 |  | 0 |
| 2 | downstream | Pańskie | 21 | 400 | 10 | 36 | 16 | 53 | 16 |  | 0 |
| 3 | downstream | Pańskie | 45 | 422 | 7 | 26 | 14 | 47 | 9 |  | 0 |
| 4 | downstream | Pańskie | 24 | 434 | 7 | 28 | 11 | 49 | 9 |  | 0 |
| 5 | downstream | Pańskie | 28 | 417 | 7 | 20 | 11 | 39 | 6 |  | 1 |
| 6 | downstream | Pańskie | 21 | 397 | 6 | 7 | 3 | 32 | 2 |  | 15 |
| 7 | downstream | Pańskie | 19 | 365 | 6 | 6 | 1 | 36 | 3 |  | 54 |
| 8 | downstream | Pańskie | 13 | 402 | 6,8 | 7 | 2 | 43 | 2 |  | 68 |
| 9 | downstream | Pańskie | 20 | 436 | 5,6 | 11 | 2 | 36 | 2 |  | 73 |
| 10 | downstream | Pańskie | 17 | 373 | 8,2 | 9 | 3 | 52 | 3 |  | 127 |
| 11 | downstream | Pańskie | 22 | 433 | 5 | 11 | 1 | 39 | 2 |  | 175 |
