# Supplementary material for: Diurnal Changes of Zooplankton Community Reduction Rate at Lake Outlets and Related Environmental Factors
Source: PLoS One. 2016 Jul 8;11(7):e0158837. doi: 10.1371/journal.pone.0158837 (PMC4938256; doi:10.1371/journal.pone.0158837)
Supplement: S2 Table — All data used for analysis. (DOCX) [file pone.0158837.s002.docx]

**S2 Table. Values of light conditions and zooplankton abundance in Młyńskie lake outlet.** All data used for analysis.

| Hour | Site | Lake outlet | Benthic rotifers  (ind l^-1^) | Pelagic rotifers  (ind l^-1^) | Asplanchna  (ind l^-1^) | Small cladocerans  (ind l^-1^) | Large cladocerans  (ind l^-1^) | Nauplii  (ind l^-1^) | Copepoda  (ind l^-1^) | Illuminance  (lux) | PAR  (µmol photons m^−2^ s^−1^) |
| --- | --- | --- | --- | --- | --- | --- | --- | --- | --- | --- | --- |
| 12 | outflow | Młyńskie | 8 | 137 | 3 | 47 | 8 | 31 | 15 | 8542 |  |
| 13 | outflow | Młyńskie | 6,6 | 156 | 8 | 42 | 7 | 22 | 7 | 8657 |  |
| 14 | outflow | Młyńskie | 11 | 142 | 4 | 54 | 9 | 21 | 12 | 7246 |  |
| 15 | outflow | Młyńskie | 5 | 178 | 5 | 51 | 9 | 36 | 7 | 7054 |  |
| 16 | outflow | Młyńskie | 7 | 263 | 5 | 47 | 8 | 42 | 11 | 5575 |  |
| 17 | outflow | Młyńskie | 5 | 272 | 4 | 76 | 6 | 47 | 16 | 3765 |  |
| 18 | outflow | Młyńskie | 6 | 332 | 7 | 94 | 11 | 62 | 12 | 3421 |  |
| 19 | outflow | Młyńskie | 11 | 504 | 6 | 154 | 15 | 77 | 15 | 1534 |  |
| 20 | outflow | Młyńskie | 7 | 543 | 16 | 272 | 13 | 76 | 13 | 467 |  |
| 21 | outflow | Młyńskie | 18 | 814 | 18 | 288 | 17 | 105 | 16 | 43 |  |
| 22 | outflow | Młyńskie | 27 | 862 | 15,4 | 411 | 42 | 122 | 31 | 0,1 |  |
| 23 | outflow | Młyńskie | 14 | 951 | 16,5 | 556 | 51 | 138 | 37 | 0 |  |
| 0 | outflow | Młyńskie | 15 | 932 | 15,2 | 572 | 55 | 135 | 31 | 0 |  |
| 1 | outflow | Młyńskie | 9 | 944 | 12 | 488 | 28 | 154 | 40 | 0 |  |
| 2 | outflow | Młyńskie | 15 | 633 | 12,5 | 316 | 35 | 133 | 27 | 0 |  |
| 3 | outflow | Młyńskie | 9 | 317 | 7,7 | 182 | 16 | 115 | 22 | 0 |  |
| 4 | outflow | Młyńskie | 11 | 345 | 7,4 | 98 | 18 | 121 | 24 | 0,2 |  |
| 5 | outflow | Młyńskie | 7 | 217 | 6,8 | 84 | 14 | 92 | 13 | 24 |  |
| 6 | outflow | Młyńskie | 21 | 232 | 4,2 | 38 | 9 | 106 | 8 | 2091 |  |
| 7 | outflow | Młyńskie | 9 | 216 | 5,5 | 28 | 5 | 117 | 12 | 4325 |  |
| 8 | outflow | Młyńskie | 11 | 132 | 5,9 | 31 | 7 | 92 | 12 | 5567 |  |
| 9 | outflow | Młyńskie | 6 | 144 | 4,8 | 45 | 11 | 74 | 15 | 6230 |  |
| 10 | outflow | Młyńskie | 8 | 156 | 3,6 | 29 | 5 | 78 | 11 | 8232 |  |
| 11 | outflow | Młyńskie | 11 | 127 | 5,1 | 55 | 6,2 | 65 | 7 | 8458 |  |
| 12 | downstream | Młyńskie | 4 | 117 | 1 | 17 | 0 | 26 | 1 |  | 319 |
| 13 | downstream | Młyńskie | 8 | 128 | 4 | 12 | 1 | 17 | 0 |  | 343 |
| 14 | downstream | Młyńskie | 8 | 125 | 1 | 15 | 0 | 17 | 2 |  | 293 |
| 15 | downstream | Młyńskie | 7 | 155 | 3 | 12 | 1 | 31 | 1 |  | 235 |
| 16 | downstream | Młyńskie | 11 | 225 | 3 | 17 | 1 | 34 | 3 |  | 213 |
| 17 | downstream | Młyńskie | 13 | 243 | 2 | 27 | 0 | 38 | 4 |  | 146 |
| 18 | downstream | Młyńskie | 16 | 302 | 5 | 29 | 1 | 55 | 3 |  | 129 |
| 19 | downstream | Młyńskie | 15 | 438 | 4 | 76 | 3 | 63 | 4 |  | 116 |
| 20 | downstream | Młyńskie | 6 | 502 | 10 | 118 | 4 | 62 | 3 |  | 24 |
| 21 | downstream | Młyńskie | 16 | 719 | 14 | 149 | 8 | 88 | 8 |  | 2 |
| 22 | downstream | Młyńskie | 19 | 745 | 13 | 321 | 27 | 103 | 21 |  | 0 |
| 23 | downstream | Młyńskie | 8 | 794 | 12,1 | 395 | 32 | 113 | 24 |  | 0 |
| 0 | downstream | Młyńskie | 23 | 880 | 13,8 | 504 | 41 | 108 | 21 |  | 0 |
| 1 | downstream | Młyńskie | 11 | 868 | 8 | 396 | 24 | 127 | 28 |  | 0 |
| 2 | downstream | Młyńskie | 10 | 547 | 10,2 | 268 | 25 | 112 | 22 |  | 0 |
| 3 | downstream | Młyńskie | 12 | 281 | 7 | 166 | 12 | 108 | 15 |  | 0 |
| 4 | downstream | Młyńskie | 15 | 291 | 6,6 | 62 | 11 | 89 | 14 |  | 0 |
| 5 | downstream | Młyńskie | 12 | 185 | 4,3 | 34 | 5 | 74 | 6 |  | 1 |
| 6 | downstream | Młyńskie | 17 | 209 | 2,8 | 11 | 1 | 87 | 2 |  | 88 |
| 7 | downstream | Młyńskie | 7 | 184 | 3,1 | 9 | 0 | 87 | 2 |  | 99 |
| 8 | downstream | Młyńskie | 11 | 117 | 2,9 | 11 | 0 | 82 | 3 |  | 188 |
| 9 | downstream | Młyńskie | 16 | 121 | 2,7 | 13 | 1 | 63 | 2 |  | 203 |
| 10 | downstream | Młyńskie | 12 | 137 | 2 | 11 | 0 | 65 | 1 |  | 265 |
| 11 | downstream | Młyńskie | 9 | 115 | 2,4 | 18 | 1 | 52 | 0 |  | 312 |
