# Supplementary material for: Diurnal Changes of Zooplankton Community Reduction Rate at Lake Outlets and Related Environmental Factors
Source: PLoS One. 2016 Jul 8;11(7):e0158837. doi: 10.1371/journal.pone.0158837 (PMC4938256; doi:10.1371/journal.pone.0158837)
Supplement: S3 Table — All data used for analysis. (DOCX) [file pone.0158837.s003.docx]

**S3 Table. Values of light conditions and zooplankton abundance in Dominikowo lake outlet.** All data used for analysis.

| Hour | Site | Lake outlet | Benthic rotifers  (ind l^-1^) | Pelagic rotifers  (ind l^-1^) | Asplanchna  (ind l^-1^) | Small cladocerans  (ind l^-1^) | Large cladocerans  (ind l^-1^) | Nauplii  (ind l^-1^) | Copepoda  (ind l^-1^) | Illuminance  (lux) | PAR  (µmol photons m^−2^ s^−1^) |
| --- | --- | --- | --- | --- | --- | --- | --- | --- | --- | --- | --- |
| 12 | outflow | Dominikowo | 2 | 42 | 0,4 | 1,6 | 0,8 | 13,6 | 1,2 | 8870 |  |
| 13 | outflow | Dominikowo | 2,4 | 50 | 0 | 2 | 1,2 | 12,4 | 2 | 5310 |  |
| 14 | outflow | Dominikowo | 1,6 | 66,4 | 0,4 | 0,8 | 1,6 | 14,4 | 1,6 | 4370 |  |
| 15 | outflow | Dominikowo | 3,2 | 112,8 | 0,4 | 0,4 | 1,2 | 31,6 | 2,4 | 4010 |  |
| 16 | outflow | Dominikowo | 0,8 | 147,2 | 0 | 2,4 | 3,6 | 50,4 | 3,6 | 3260 |  |
| 17 | outflow | Dominikowo | 2,8 | 339,2 | 0 | 2,8 | 16,4 | 95,2 | 9,6 | 2590 |  |
| 18 | outflow | Dominikowo | 2 | 347,2 | 0 | 4,4 | 17,2 | 93,2 | 5,6 | 2090 |  |
| 19 | outflow | Dominikowo | 1,2 | 365,2 | 0,4 | 6 | 20,4 | 107,2 | 8 | 940 |  |
| 20 | outflow | Dominikowo | 2 | 361,2 | 0 | 4 | 40,4 | 126,4 | 12 | 390 |  |
| 21 | outflow | Dominikowo | 2,4 | 272 | 0,8 | 6 | 22 | 105,6 | 15,2 | 0,1 |  |
| 22 | outflow | Dominikowo | 0,4 | 292,4 | 1,2 | 2,8 | 18,8 | 88 | 14,4 | 0 |  |
| 23 | outflow | Dominikowo | 0 | 302,4 | 1,6 | 2,4 | 22 | 84,4 | 23,6 | 0 |  |
| 0 | outflow | Dominikowo | 0,4 | 171,2 | 2,4 | 3,2 | 14,4 | 72,8 | 24,8 | 0 |  |
| 1 | outflow | Dominikowo | 0,8 | 180,8 | 5,2 | 2 | 16,4 | 59,6 | 28,8 | 0 |  |
| 2 | outflow | Dominikowo | 1,6 | 63,2 | 1,6 | 1,2 | 10,4 | 31,2 | 10,8 | 0 |  |
| 3 | outflow | Dominikowo | 1,6 | 36,4 | 0 | 0 | 2,8 | 14,8 | 4,4 | 0 |  |
| 4 | outflow | Dominikowo | 2,8 | 26,4 | 0 | 0 | 2,4 | 19,2 | 4 | 0,01 |  |
| 5 | outflow | Dominikowo | 6 | 26 | 0 | 0,4 | 0,8 | 16,8 | 2 | 0,08 |  |
| 6 | outflow | Dominikowo | 1,6 | 36,4 | 0 | 0,4 | 1,2 | 14 | 2 | 850 |  |
| 7 | outflow | Dominikowo | 1,2 | 34,4 | 0 | 0 | 0,8 | 15,2 | 1,2 | 3890 |  |
| 8 | outflow | Dominikowo | 2,8 | 69,6 | 0,4 | 2,4 | 1,2 | 22,8 | 1,2 | 5200 |  |
| 9 | outflow | Dominikowo | 2,4 | 82,8 | 0 | 3,2 | 1,2 | 22 | 2,4 | 5800 |  |
| 10 | outflow | Dominikowo | 5,6 | 75,2 | 0 | 2,4 | 3,2 | 17,2 | 2,8 | 7580 |  |
| 11 | outflow | Dominikowo | 1,6 | 40 | 0 | 1,2 | 0 | 11,2 | 0,8 | 9670 |  |
| 12 | downstream | Dominikowo | 2,8 | 35,6 | 0 | 0,4 | 0 | 12 | 0 |  | 407 |
| 13 | downstream | Dominikowo | 2 | 42,8 | 0 | 0 | 0 | 11,6 | 0,4 |  | 275 |
| 14 | downstream | Dominikowo | 4 | 56,4 | 0 | 0 | 0 | 12,4 | 0,4 |  | 151 |
| 15 | downstream | Dominikowo | 4 | 95,2 | 0 | 0 | 0 | 21,6 | 0,4 |  | 140 |
| 16 | downstream | Dominikowo | 2,8 | 133,2 | 0 | 0,8 | 0 | 44,4 | 0,4 |  | 115 |
| 17 | downstream | Dominikowo | 3,6 | 287,6 | 0 | 0,4 | 0,8 | 57,2 | 0 |  | 105 |
| 18 | downstream | Dominikowo | 3,2 | 313,2 | 0 | 1,2 | 1,2 | 84,8 | 0,4 |  | 96 |
| 19 | downstream | Dominikowo | 4,4 | 328,4 | 0 | 2 | 0,8 | 96,4 | 1,6 |  | 56 |
| 20 | downstream | Dominikowo | 5,6 | 312,4 | 0 | 0,8 | 2,8 | 110,8 | 4 |  | 17 |
| 21 | downstream | Dominikowo | 7,2 | 246,8 | 0,4 | 3,6 | 10,8 | 94,8 | 8,4 |  | 0 |
| 22 | downstream | Dominikowo | 1,6 | 251,2 | 0,8 | 1,6 | 11,2 | 73,6 | 8 |  | 0 |
| 23 | downstream | Dominikowo | 0 | 249,2 | 0,8 | 2 | 11,6 | 69,2 | 13,6 |  | 0 |
| 0 | downstream | Dominikowo | 2 | 152,8 | 1,6 | 2,4 | 9,6 | 63,6 | 16,8 |  | 0 |
| 1 | downstream | Dominikowo | 2 | 166,8 | 4 | 1,2 | 10 | 55,2 | 16,8 |  | 0 |
| 2 | downstream | Dominikowo | 4 | 54 | 1,2 | 1,2 | 6,8 | 26 | 6,4 |  | 0 |
| 3 | downstream | Dominikowo | 3,6 | 33,6 | 0 | 0 | 2 | 11,6 | 3,2 |  | 0 |
| 4 | downstream | Dominikowo | 3,2 | 22,8 | 0 | 0 | 1,2 | 16,4 | 2,4 |  | 0 |
| 5 | downstream | Dominikowo | 6,4 | 22,4 | 0 | 0 | 0,4 | 15,2 | 1,2 |  | 0 |
| 6 | downstream | Dominikowo | 3,6 | 30,8 | 0 | 0 | 0 | 10,4 | 0,4 |  | 44 |
| 7 | downstream | Dominikowo | 2,8 | 28,4 | 0 | 0 | 0 | 12,4 | 0 |  | 137 |
| 8 | downstream | Dominikowo | 4 | 61,2 | 0 | 0,8 | 0,4 | 18,8 | 0,4 |  | 211 |
| 9 | downstream | Dominikowo | 3,6 | 73,6 | 0 | 0,4 | 0 | 19,2 | 0,4 |  | 262 |
| 10 | downstream | Dominikowo | 6,8 | 65,2 | 0 | 0,4 | 0 | 15,2 | 0,4 |  | 378 |
| 11 | downstream | Dominikowo | 4,8 | 34 | 0 | 0 | 0 | 9,6 | 0 |  | 634 |
