# Supplementary material for: Diurnal Changes of Zooplankton Community Reduction Rate at Lake Outlets and Related Environmental Factors
Source: PLoS One. 2016 Jul 8;11(7):e0158837. doi: 10.1371/journal.pone.0158837 (PMC4938256; doi:10.1371/journal.pone.0158837)
Supplement: S4 Table — All data used for analysis. (DOCX) [file pone.0158837.s004.docx]

**S4 Table. Values of light conditions and zooplankton abundance in Korytnica lake outlet.** All data used for analysis.

| Hour | Site | Lake outlet | Benthic rotifers  (ind l^-1^) | Pelagic rotifers  (ind l^-1^) | Asplanchna  (ind l^-1^) | Small cladocerans  (ind l^-1^) | Large cladocerans  (ind l^-1^) | Nauplii  (ind l^-1^) | Copepoda  (ind l^-1^) | Illuminance  (lux) | PAR  (µmol photons m^−2^ s^−1^) |
| --- | --- | --- | --- | --- | --- | --- | --- | --- | --- | --- | --- |
| 12 | outflow | Korytnica | 6 | 221 | 2,8 | 24,2 | 11 | 82,5 | 38 | 7863 |  |
| 13 | outflow | Korytnica | 4 | 216 | 2,2 | 19 | 15 | 88,6 | 31 | 7638 |  |
| 14 | outflow | Korytnica | 5 | 234 | 0,8 | 31 | 12 | 97 | 33 | 6425 |  |
| 15 | outflow | Korytnica | 7 | 278 | 1,5 | 17 | 9 | 86 | 35 | 6213 |  |
| 16 | outflow | Korytnica | 6 | 268 | 3,6 | 22,6 | 14 | 82 | 41 | 5237 |  |
| 17 | outflow | Korytnica | 8 | 486 | 3,2 | 28 | 12 | 104 | 49 | 3478 |  |
| 18 | outflow | Korytnica | 14 | 573 | 3,8 | 34 | 15 | 98 | 56 | 3041 |  |
| 19 | outflow | Korytnica | 8 | 744 | 6,6 | 52 | 22 | 122 | 54 | 1423 |  |
| 20 | outflow | Korytnica | 5 | 721 | 10,4 | 58 | 28 | 156 | 72 | 550 |  |
| 21 | outflow | Korytnica | 8 | 758 | 12,2 | 64 | 24 | 116 | 80,6 | 58 |  |
| 22 | outflow | Korytnica | 11 | 905 | 18,5 | 87 | 38 | 164 | 122,2 | 0,1 |  |
| 23 | outflow | Korytnica | 5 | 1231 | 17,4 | 121 | 52 | 158,2 | 135,5 | 0 |  |
| 0 | outflow | Korytnica | 7 | 1024 | 18,8 | 134 | 43 | 188,4 | 127 | 0 |  |
| 1 | outflow | Korytnica | 11 | 1233 | 16,4 | 138 | 32 | 224 | 95 | 0 |  |
| 2 | outflow | Korytnica | 4 | 833 | 12,6 | 122 | 34 | 182 | 74 | 0 |  |
| 3 | outflow | Korytnica | 7 | 504 | 8,8 | 84 | 25 | 108,6 | 62,4 | 0 |  |
| 4 | outflow | Korytnica | 6 | 458 | 6,2 | 42 | 15 | 115,6 | 32 | 0,4 |  |
| 5 | outflow | Korytnica | 10 | 281 | 4,4 | 30 | 17 | 105 | 35 | 31 |  |
| 6 | outflow | Korytnica | 6 | 309 | 1,6 | 21 | 14 | 82 | 24 | 1476 |  |
| 7 | outflow | Korytnica | 4 | 276 | 2,2 | 24 | 14 | 88,8 | 32 | 4581 |  |
| 8 | outflow | Korytnica | 7 | 298 | 2,8 | 22,4 | 11 | 70,5 | 36 | 5886 |  |
| 9 | outflow | Korytnica | 5 | 264 | 3,2 | 18,6 | 16 | 84,7 | 28 | 6458 |  |
| 10 | outflow | Korytnica | 11 | 283 | 2,4 | 24,8 | 8 | 78,6 | 42 | 7743 |  |
| 11 | outflow | Korytnica | 6 | 288 | 2,5 | 26,7 | 10 | 70,4 | 26 | 8883 |  |
| 12 | downstream | Korytnica | 14 | 197 | 1,5 | 11 | 4 | 70,1 | 8 |  | 197 |
| 13 | downstream | Korytnica | 12 | 205 | 2 | 11 | 3 | 75,1 | 12 |  | 171 |
| 14 | downstream | Korytnica | 18 | 214 | 0,6 | 11 | 2 | 80 | 8 |  | 163 |
| 15 | downstream | Korytnica | 23 | 235 | 1,4 | 5,7 | 1 | 64 | 9 |  | 161 |
| 16 | downstream | Korytnica | 15 | 242 | 2,5 | 7 | 2 | 72 | 8 |  | 124 |
| 17 | downstream | Korytnica | 12 | 432 | 2,6 | 12 | 3 | 85 | 14 |  | 84 |
| 18 | downstream | Korytnica | 22 | 542 | 2,4 | 12 | 3 | 84 | 11 |  | 71 |
| 19 | downstream | Korytnica | 26 | 688 | 4,6 | 23 | 5 | 111 | 11 |  | 54 |
| 20 | downstream | Korytnica | 12 | 649 | 8,4 | 28 | 11 | 127,6 | 25 |  | 21 |
| 21 | downstream | Korytnica | 31 | 702 | 10 | 39 | 17 | 107 | 54 |  | 3 |
| 22 | downstream | Korytnica | 16 | 823 | 16,3 | 71 | 25 | 132 | 78 |  | 0 |
| 23 | downstream | Korytnica | 12 | 1188 | 15,5 | 91 | 34 | 138,5 | 89 |  | 0 |
| 0 | downstream | Korytnica | 25 | 978 | 16,2 | 104 | 27 | 168 | 82 |  | 0 |
| 1 | downstream | Korytnica | 18 | 1156 | 14,8 | 116 | 22 | 177 | 74 |  | 0 |
| 2 | downstream | Korytnica | 11 | 738 | 10,6 | 98 | 22 | 162 | 43 |  | 0 |
| 3 | downstream | Korytnica | 26 | 442 | 7,6 | 66 | 20 | 95 | 42 |  | 0 |
| 4 | downstream | Korytnica | 15 | 376 | 5,2 | 22 | 8 | 102 | 19 |  | 0 |
| 5 | downstream | Korytnica | 16 | 245 | 3 | 14 | 11 | 94 | 12 |  | 1 |
| 6 | downstream | Korytnica | 11 | 267 | 1 | 7 | 4 | 67 | 7 |  | 58 |
| 7 | downstream | Korytnica | 9 | 244 | 2 | 11 | 2 | 80 | 4 |  | 79 |
| 8 | downstream | Korytnica | 17 | 267 | 1,6 | 9 | 3 | 62 | 5 |  | 152 |
| 9 | downstream | Korytnica | 9 | 242 | 2,4 | 11 | 3 | 78 | 6 |  | 165 |
| 10 | downstream | Korytnica | 25 | 251 | 2 | 12 | 2 | 70 | 10 |  | 186 |
| 11 | downstream | Korytnica | 13 | 268 | 2 | 10 | 3 | 65 | 4 |  | 248 |
